# Laparoscopic Sleeve Gastrectomy as a Primary Operation for Morbid Obesity: Experience with 200 Patients

**DOI:** 10.1155/2012/801325

**Published:** 2012-06-03

**Authors:** Paolo Gentileschi

**Affiliations:** Bariatric Surgery Unit, University of Rome Tor Vergata, Rome, Via A. Bosio 13, 00161 Rome, Italy

## Abstract

*Introduction*. Laparoscopic sleeve gastrectomy (LSG) represents a valid option for morbid obesity, either as a primary or as a staged procedure. The aim of this paper is to report the experience of a single surgeon with LSG as a standalone operation for morbid obesity. *Methods*. From April 2006 to April 2011, 200 patients underwent LSG for morbid obesity. Each patient record was registered and prospectively collected. In July 2011, a retrospective analysis was conducted. *Results*. Patients were 128 females and 72 males with a median age of 40.0 years. Median pre-operative BMI was 49.4 kg/m^2^. Median follow-up was 27.2 months. Median post-operative BMI was 30.4 kg/m^2^. Median %excess weight loss (%EWL) was 63.6%. Median post-operative hospital stay was 4.0 days in the first 84 cases and 3.0 days in the last 116 cases. Six major post-operative complications occurred (3%): two gastric stump leaks (1%), three major bleedings (1.5%) and 1 (0.5%) bowel obstruction. One case of mortality was registered (0.5%). To date only 4 patients are still in the range of morbid obesity (BMI > 35 kg/m^2^). *Conclusions*. Laparoscopic sleeve gastrectomy is a formidable operation in the short-term period. Median %EWL in this series was 63.6% at 27.2 months follow-up.

## 1. Introduction

Obesity is a major worldwide problem in public health, reaching epidemic proportions in western countries. Bariatric surgery has been shown to be more effective in the management of morbid obesity, compared to medical treatments in terms of weight loss and amelioration of comorbidities [1].

Laparoscopic sleeve gastrectomy (LSG) represents a valid option for morbidly obese patients, either as a primary or as a staged bariatric procedure. Initially, LSG was conceived as a restrictive component of the biliopancreatic diversion and duodenal switch. Later on, LSG has been proposed as a step procedure in high-risk patients, followed by a second step Roux-en-Y gastric bypass or biliopancreatic diversion and duodenal switch [2]. Recently, LSG has been proposed as a standalone bariatric procedure. Excess weight loss and remission of comorbidities have been reported to take place in a frequency comparable with other well-established procedures [3].

The experience at our institution with LSG as a primary operation for morbid obesity started in 2006. The aim of this work is to report the five-year experience of a single surgeon single center with LSG as a sole operation for morbid obesity.

## 2. Methods

At our Institution, the bariatric surgery program started in 1996, with a laparoscopic gastric banding. After a 10-year experience with laparoscopic Roux-en-Y gastric bypass, gastric banding, biliopancreatic diversion with duodenal switch, and several bariatric reoperations, the first LSG was performed in April 2006. From April 2006 to April 2011, 200 patients were submitted to LSG as a primary procedure for morbid obesity.

Patients were studied preoperatively with a multi-disciplinary workup including specialistic counseling (surgery, endocrinology, internal medicine, psychiatry, anesthesiology), gastrointestinal endoscopy, and complete performance status evaluation. Patients were well informed about the surgical procedure, with all potential advantages and possible complications and side effects. Indications for LSG were the following conditions: BMI over 60 kg/m^2^ in high-risk patients, BMI between 50 and 60 kg/m^2^ in nondiabetic patients, BMI between 40 and 50 kg/m^2^ in patients refusing a complex procedure like Roux-en-Y gastric bypass, patients with severe gastric inflammatory disease and *H. pylori* infection, patients with previous abdominal surgery involving the intestines, and young patients refusing gastric banding. Diabetic patients were first selected for Roux-en-Y gastric bypass and then to LSG if they refused the gastric bypass. Specific contra-indications, apart from the general contraindications to bariatric surgery, were severe and documented gastroesophageal reflux disease and previous gastric surgery.

**Figure 1:**
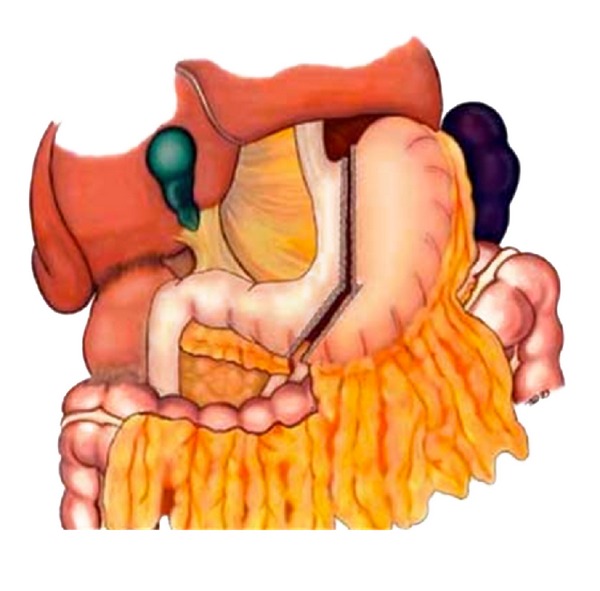
Surgical technique of sleeve gastrectomy.

Standard surgical technique (Figure 1) was as follows: the patient was placed in a supine position on the operating table with his arms extended in abduction and legs opened, in reverse Trendelenburg position with a 10° tilt. The surgeon stood between the legs with the assistant on the left side of the patient, and the cameraperson to the patient's right. Abdominal insufflation was set at 15 mmHg. Trocars were placed as follows: a 10 mm trocar (T1) 20 cm below the xiphoid process for the 30° optical system, a 5 mm trocar (T2) on the left anterior axillary line, a 12 mm trocar (T3) on the left mid-clavicular line just between the first and the second trocars, a 12 mm trocar (T4) on the right midclavicular line, and a 5 mm trocar (T5) below the xiphoid process. Using a dissecting coagulator (Ultracision, Ethicon Endo-Surgery), the greater curvature of the stomach was mobilized at a point 3 cm proximal to the pylorus. The lesser sac was entered, and staying close to the wall of the stomach, the greater curvature ligaments (gastrosplenic and gastrocolic) were divided all the way up to the angle of His.

We paid particular attention to the identification and mobilization of the angle of His with exposure of the left crus of the diaphragm to delineate the gastroesophageal junction and to facilitate complete resection of the gastric fundus. Retrogastric adhesions were taken down with the Ultracision device to allow for complete mobilization of the stomach, to eliminate any redundant posterior wall of the sleeve, and to exclude the fundus from the gastric sleeve.

After a complete mobilization of the stomach was reached, a 40 Fr orogastric tube was inserted transorally into the pylorus and placed against the lesser curvature. This helped to calibrate the size of the gastric sleeve, prevent any constriction at the gastroesophageal junction, and provide a uniform shape to the entire stomach.

Gastric transection by a cutting stapler (recently, a Echelon Flex, Ethicon Endo-Surgery) began at a point 3 cm proximal to the pylorus, leaving the antrum and preserving gastric emptying. The stapler was fired consecutively along the length of the orogastric tube until the angle of His was reached. Care was taken not to narrow the stomach at the angularis. We considered important to inspect the stomach anteriorly and posteriorly to ensure no redundant posterior stomach.

The entire staple line was inspected for bleeding and tested for leak. The patient was placed flat, and an atraumatic clamp was placed near the pylorus. The integrity of the staple line was tested by insufflating air under saline and infusing 30–60 cm³ of methylene blue into the remaining stomach.

Staple line reinforcement was performed using various techniques: (1) the stapler was supported by the application of the Gore Seamguard (W. L. Gore & Associates, Flagstaff, AZ, USA) before the introduction of the device in the abdomen; (2) the staple line was reinforced by seroserosal running sutures using absorbable material from the last firing of the stapler towards the first one; (3) the entire staple line was covered by Floseal, a thrombin gelatin matrix, gently squeezed over the entire staple line. The resected stomach was extracted through the periumbilical incision at the end of the procedure. At the beginning of the experience a nasogastric tube and a suction drain were left in place; recently, no drains and nasogastric tube were used. The fascial defects were closed with a figure of eight 2/0 nonabsorbable suture to prevent port site hernia.

**Figure 2:**
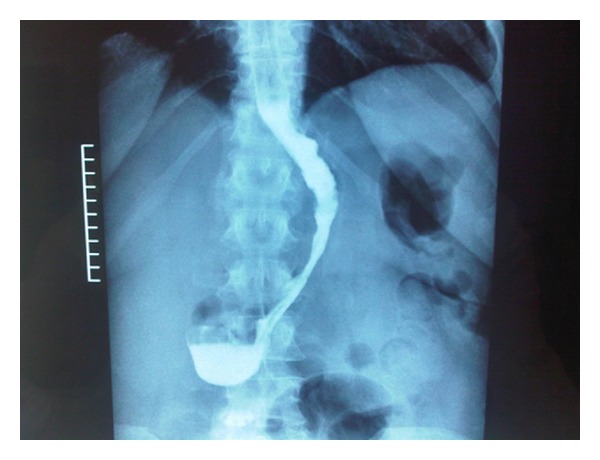
Postoperative swallow test.

A gastrografin swallow test was performed on the first postoperative day (Figure 2), and a liquid diet was started. Patients were discharged from the hospital as soon as they could walk, drink, with no fever or any clinical complication. Patients were given a liquid diet for two weeks and followed up in our outpatient clinic for years. Followup was performed by an endocrinologist, a nutrition expert, surgeon, and psychiatrist. The frequency of each outpatient visit was every three months for the first year and every six months for the following years.

Each patient record was registered by the surgical team and prospectively collected. In July 2011, a retrospective analysis was conducted and results are hereby presented.

## 3. Results

From April 2006 to April 2011, 200 patients underwent a laparoscopic sleeve gastrectomy as a primary operation for morbid obesity. They were 128 females and 72 males with a median age of 40.0 years (range from 16 to 64 years). Median preoperative BMI was 49.4 kg/m^2^ (range from 40 to 78 kg/m^2^). Median followup was 27.2 months (range from 3 to 63 months). Followup last update was performed successfully in all patients, in 182 cases with a hospital visit and in 17 cases by phone (1 patient died postoperatively). Median postoperative BMI at last followup visit was 30.4 kg/m^2^ (range from 25 to 37 kg/m^2^). Median % excess weight loss (% EWL) at last followup visit was 63.6%.

Median postoperative hospital stay was 4.0 days (range from 3 to 62 days) in the first 84 cases and 3.0 days (range from 3 to 28 days) in the last 116 cases. This result was achieved with a new protocol involving prehospitalization, admission on the day of surgery, and early discharge. Median operative time was 102 minutes (range from 64 to 180 minutes). Conversion to open surgery was required in 2 cases (1%) for difficult dissection in 2 patients with previous abdominal surgery.

Six major postoperative complications were observed (3%). Two gastric stump leaks (1%), three major bleedings (1.5%), and 1 (0.5%) small bowel obstruction occurred. The two gastric leaks occurred both at the gastroesophageal junction. One case of mortality was registered on the 62nd postoperative day (0.5%).

The first leak was in a 16-year-old girl with a preoperative BMI of 68 kg/m^2^ submitted to LSG in April 2009. Staple-line reinforcement was performed with the thrombin matrix. After an uneventful procedure and a regular postoperative course, the patient was discharged on the 5th postoperative day. She was readmitted on the 9th postoperative day with abdominal pain and fever. CT scan with a gastrografin swallow revealed a small leak from the gastric stump immediately below the gastroesophageal junction. She was treated with relaparoscopy, drainage, and total parenteral nutrition. A new swallow two weeks later showed complete healing of the gastric fistula, and oral feeding was started. She is doing fine at two-year followup.

The second leak occurred in a 52-year-old man with a preoperative BMI of 58 kg/m^2^. He underwent a LSG with a staple line reinforcement performed with a running suture. The patient had severe comorbidities including type II diabetes, sleep apnea, hypertension, and mild renal failure. He was discharged on the 5th postoperative day on a liquid diet after a regular swallow test. He was readmitted on the 8th postoperative day with abdominal pain, fever, and renal failure. CT scan with a gastrografin swallow showed a large (approximately 1.5 cm) gastric stump leak at the gastroesophageal junction with peritonitis. He underwent relaparoscopy, suture of the defect, drainage, and total parenteral nutrition. Unfortunately his postoperative course was complicated by renal failure and bilateral pneumonia requiring intensive care stay. Although a CT scan revealed no further abdominal complications, the patient died on the 62nd postoperative day. At autopsy, a severe bilateral pneumonia was diagnosed with no abdominal infection.

Three cases of major bleeding occurred (1.5%), two from the staple line and one from the trocar site. The two cases of bleeding from the staple line were observed after reinforcement performed in the first case with a running suture and in the second case with Seamguard. They were treated with laparoscopic exploration and suture of the bleeding vessels. The patient with bleeding from the trocar wound was also treated by laparoscopic exploration and open suture of the left epigastric artery. One blood transfusion was required in the last patient. One small bowel obstruction occurred in a patient submitted to LSG and discharged on the 3rd postoperative day. She underwent a laparotomy in another hospital, and surgeons found an acute hernia from a trocar incision with small bowel obstruction.

With a median followup of 27.2 months, a median post-operative BMI of 30.4 kg/m^2^  was registered. Apart from 12 patients who have a short followup (less than 6 months), to date, only 4 patients are still in the range of morbid obesity (BMI > 35 kg/m^2^). Comorbidity resolution and improvement were seen in type II diabetes, hypertension, and obstructive sleep apnea (Table 1).

## 4. Discussion

Bariatric surgery is the only evidence-based approach to sustainable weight loss, improving comorbid disease and survival in morbidly obese patients.

Laparoscopic sleeve gastrectomy is a formidable operation in terms of weight loss in the short and mid-term period (5 years). As a two-stage procedure, LSG was initially performed using a 60-French bougie with a 33% excess weight loss at 11 months [4]. Since then surgeons began using LSG as a primary procedure using smaller-sized bougies with greater % EWL (62%) [5]. In a recent systematic review, weight loss ranged from 33 to 85% of excess weight in patients submitted to LSG [6]. The durability of LSG at 5 years has been clearly demonstrated [7]. Mean % EWL in this series was 64.4% at 28.2-month followup. Clinical long-term results of LSG will emerge in the future when most of the authors report their experience with primary LSG over a period superior to 10 years. To date, we must conclude that LSG is an excellent weight loss operation in the short and mid-term period. In addition, LSG seems to have the potential to be an effective standalone procedure for durable weight loss and comorbid resolution. Doubts still exist regarding weight regain or the desire for further weight loss in the superobese patients requiring the addition of a gastric bypass or biliopancreatic diversion to LSG. Furthermore, there is continued disagreement as to whether LSG represents a restrictive procedure versus a combination restrictive/hormonal procedure. Studies have shown the effects of LSG on ghrelin levels and hunger [8], as well as additional metabolic hormones [9].

Laparoscopic sleeve gastrectomy seems to offer certain advantages compared to well-established procedures like gastric banding, Roux-en-Y gastric bypass, or biliopancreatic diversion: lack of an intestinal anastomosis, normal intestinal absorption, no risk of internal hernias, no implantation of a foreign body, pylorus preservation with no dumping syndrome, continuity of gastrointestinal continuity with the possibility to explore the entire gastrointestinal tract. Main concerns of LSG remain unclear and need further investigation, like the risk of important postoperative complications including staple-line leak (1–3%) and postoperative hemorrhage (3.5%).

In this series in 200 consecutive cases, 6 major complications occurred (3%). Of the two gastric stump leaks, one was diagnosed two weeks after the operation and was successfully managed by laparoscopic drainage and total parenteral nutrition with complete healing of the gastric fistula. The patient is doing well at two-year followup. Unfortunately, the other patient with a greater leak died two months after the operation.

Some considerations must be done about this case. He was a high-risk patient with a BMI of 58 kg/m^2^ with severe comorbidities. Gastric leak probably occurred one week after the operation and was treated by reoperation with laparoscopic suture and drainage. At necropsy, no gastric fistula or peritonitis was diagnosed and the patient died of bilateral pneumonia and renal failure.

A mortality rate of 0.5% in a high-risk group of patients is acceptable, nevertheless the treatment of gastric leaks after sleeve gastrectomy needs further investigation. To date, most of the authors believe that the gold standard approach for early gastric leaks is percutaneous drainage, total parenteral nutrition, antibiotic therapy, and observation [10]. When gastric fistula occurs in the 1st or 2nd postoperative day a laparoscopic approach with direct suture is justified and has potential advantages. In the two cases presented in this series, both leaks occurred in the second postoperative week and were treated by laparoscopic exploration and drainage, with suture of the defect in the second case.

In conclusion, LSG is a safe and effective primary treatment for morbid obesity at mid-term followup. In the present single surgeon single center experience, LSG is associated with excellent weight loss results with a median followup of 27.2 months in a various population of morbidly obese patients. Long-term studies are necessary in order to assess the exact role of sleeve gastrectomy in the world of bariatric surgery.

## Figures and Tables

**Table 1:** Comorbidity resolution.

Comorbidity	Improved	Resolved
(1) Diabetes (*n* = 8; 4%)	1 (12.5%)	7 (87.5%)
(2) Hypertension (*n* = 98; 49%)	26 (26.5%)	72 (73.5%)
(3) Obstructive sleep apnea (*n* = 64; 32%)	12 (18.7%)	52 (81.3%)
